# Engineered red Opto-mGluR6 Opsins, a red-shifted optogenetic excitation tool, an in vitro study

**DOI:** 10.1371/journal.pone.0311102

**Published:** 2024-10-24

**Authors:** Hoda Shamsnajafabadi, Zahra-Soheila Soheili, Mehdi Sadeghi, Shahram Samiee, Pouria Ghasemi, Mohammad Ismail Zibaii, Hamid Gholami Pourbadie, Hamid Ahmadieh, Ehsan Ranaei Pirmardan, Najmeh Salehi, Dorsa Samiee, Ali Kashanian

**Affiliations:** 1 Department of Molecular Medicine, National Institute of Genetic Engineering and Biotechnology (NIGEB), Tehran, Iran; 2 Blood Transfusion Research Center, High Institute for Research and Education in Transfusion Medicine, Tehran, Iran; 3 Laser & Plasma Research Institute, Shahid Beheshti University, Tehran, Iran; 4 Department of Physiology and Pharmacology, Pasteur Institute of Iran (IPI), Tehran, Iran; 5 Ophthalmic Research Center, Research Institute for Ophthalmology and Vision Science, Shahid Beheshti University of Medical Sciences, Tehran, Iran; 6 Molecular Biomarkers Nano-imaging Laboratory, Brigham & Women’s Hospital, Department of Radiology, Harvard Medical School, Boston, Massachusetts, United States of America; 7 School of Biological Science, Institute for Research in Fundamental Sciences (IPM), Tehran, Iran; 8 Department of Computer Science, Royal Holloway University of London, Egham, Surrey, United Kingdom; Children’s Hospital Affiliated of Zhengzhou University: Zhengzhou Children’s Hospital, CHINA

## Abstract

Degenerative eye diseases cause partial or complete blindness due to photoreceptor degeneration. Optogenetic gene therapy is a revolutionary technique combining genetics and optical methods to control the function of neurons. Due to the inherent risk of photochemical damage, the light intensity necessary to activate Opto-mGluR6 surpasses the safe threshold for retinal illumination. Conversely, red-shifted lights pose a significantly lower risk of inducing such damage compared to blue lights. We designed red-shifted Opto-mGluR6 photopigments with a wide, red-shifted working spectrum compared to Opto-mGluR6 and examined their excitation capability in vitro. ROM19, ROM18 and ROM17, red-shifted variants of Opto-mGluR6, were designed by careful bioinformatics/computational studies. The predicted molecules with the best scores were selected, synthesised and cloned into the pAAV-CMV-IRES-EGFP vector. Expression of constructs was confirmed by functional assessment in engineered HEK-GIRK cells. Spectrophotometry and patch clamp experiments demonstrated that the candidate molecules were sensitive to the desired wavelengths of the light and directly coupled light stimuli to G-protein signalling. Herein, we introduce ROM17, ROM18 and ROM19 as newly generated, red-shifted variants with maximum excitation red-shifted of ~ 40nm, 70 nm and 126 nm compared to Opto-mGluR6.

## Introduction

Genetic retinal degenerative diseases, such as Leber congenital amaurosis and retinitis pigmentosa, are major causes of blindness worldwide. Several strategies are under investigation for the treatment and repair of aforesaid disorders [1, 2]. Pharmacologic [3, 4] or gene therapies [5, 6] target the disease states at a very early age and aim to delay or stop the degenerative process. Vision loss is typically only noticeable in the later stages of photoreceptor loss. An alternative strategy involves imparting light sensitivity to neurons in the inner retina, which survive following photoreceptor loss, by optogenetic gene therapy [7, 8]. Optogenetic treatment is a long-term, ambulatory treatment that restores high-resolution vision throughout the entire visual field [9–12]. A significant therapeutic potential of optogenetic gene therapy is associated with introducing opsins as light-sensitive proteins into surviving retinal cells. Channelrhodopsin-2 (ChR2), as first described microbial-type opsin, requires extremely blue light intensities to function [13]. Exposure to blue light has a significant risk of causing photochemical damage to both the sensory retina and the retinal pigment epithelium [14]. In mammals, natural chromophores like flavins, haemoglobin, and melanin absorb blue light wavelengths more effectively, leading to greater scattering of blue light compared to yellow-red wavelengths.

The low visible light transmission in organic tissues makes red-shifted ChR variants particularly important, as longer wavelength light can penetrate deeper into the tissues [15–17]. The first red-shifted Channelrhodopsin, VChR1, by a peak response at 589 nm [18], showed effective vision restoration in blind rats [19]. A recent molecular engineering study created a novel mutant of the channelrhodopsin called red-activatable Channelrhodopsin (ReaChR), which is ideally stimulated by red light wavelengths ranging from 590 to 630 nm. It exhibits enhancements in membrane trafficking, elevated photocurrents, and quicker kinetics compared to the VChR1 mutant [20]. In blind mice, macaques and humans, ReaChR was able to restore light responses at the retina [14]. More recently, Chrimson has been recognized as a natural variant boasting the most red-shifted absorption peak at 660 nm, rendering it among the most promising ChRs for both basic neuroscientific investigations as well as potential medical applications [21]. The partial vision recovery in a blind patient following Chrimon gene therapy was reported [10]. However, each of these approaches exhibits significant limitations. As the main opsins are microbial, they are alien to inner retinal cells because preexisting pathways do not regulate their activities. On the other hand, the microbial opsins represent fast kinetics and follow high-frequency modulation of light but at the same time they have a low sensitivity to the irradiated light. In contrast, eukaryotic opsins such as rhodopsin, cone opsin and melanopsin are extremely sensitive to light due to the properties of the G-protein signalling cascade, which amplifies and modulates the light response [22–24]. However, their main limitation is the slow response to the light at the extent that they may not support patterned vision [22]. To overcome this problem, Opto-mGluR6 as a chimera of the intracellular domain of the ON-bipolar cell-specific metabotropic glutamate receptor, mGluR6, and the light-sensing domains of melanopsin, was introduced [25–27]. Although Opto-mGluR6 (λm = 467 nm) restored vision in rd1 mice, it represented blue light opsin limitation such as ChR2. Herein, we introduced ROM19 (λ = 606 nm), ROM18 (λ = 550 nm) and ROM17 (λ = 520 nm) as broader, red-shifted action spectrum variants of Opto-mGluR6 (λ = 480 nm). They represented promising expression in the HEK-GIRK cell line and revealed evoked photocurrents similar to Opto-mGluR6. They red-shift variants, ROM19, ROM18 and ROM17, correspond to maximum excitation red-shifts of 40 nm, 70 nm and 126 nm, respectively, compared with Opto-mGluR6.

## Material and methods

### Computational study

#### Designing and protein structure modelling of red Opto-mGluR6

Melanopsin (λ = 467 nm) is the light-sensing domain of the Opto-mGluR6 chimaera. Toward the design of red Opto-mGluR6 variants, one hypothesis suggested that melanopsin would be replaced by native mouse rhodopsin (λmax = 498 nm), green opsin (λmax = 508 nm) and human red opsin (λmax = 558 nm). However, this hypothesis does not prove to be true since melanopsin is an exceptional bleach-resistant bistable opsin, unlike other eukaryotic opsins [28]. Retinal binding pocket amino acids in the tertiary structure of opsins have an important role in absorption spectrums [29]. An alternative hypothesis was to replace specific amino acids in the Opto-mGluR6 structure, specifically in the retinal binding pocket.

Given the lack of an experimental three-dimensional (3D) structure for opto-mGluR6, protein structure modelling was employed. The FASTA sequence of opto-mGluR6 was submitted to the SWISS-MODEL [30] (https://swissmodel.expasy.org/) and I-TASSER [31] (https://zhanggroup.org/I-TASSER/) web servers as an input, without assigning any restraints or templates. The SWISS-MODEL and I-TASSER web servers predict the 3D structure of proteins based on homology modelling and protein threading, respectively. The best-predicted models with the highest confidence in both methods were compared. The most similar structure between these methods, with the lowest RMSD value, was selected for further analysis. The quality of the structural model of opto-mGluR6 was verified by MolProbity [32]. The Molegro Virtual Docker [33] was used to find the chromophore binding site (retinal binding pocket) in the melanopsin model.

In the next step, natural melanopsins, rhodopsin, blue, green, and red opsins sequences were retrieved from NCBI. Opsins protein sequences were sorted based on their maximum absorption. Then multiple sequence alignments (MSA) were performed and represented by the WebLogo server (http://weblogo.berkeley.edu/logo.cgi). The MSA revealed similar mutations in the retinal binding pocket of naturally occurring red opsins, which are recruited to design red-shifted variants of Opto-mGluR6.

The designed sequences were submitted to the RNAfold web server (http://rna.tbi.univie.ac.at/cgi-bin/RNAWebSuite/RNAfold.cgi) [34] to consider the RNA folding and stability. The best candidates of red-shifted variants and Opto-mGluR6 (as control) were applied in the following computational and experimental analysis.

The psfgen plugin in VMD1.9.3 [35] was used to model the three designed forms of red opto-mGluR6 variants (ROM17, ROM18, and ROM19) based on the predicted structural model of wild-type opto-mGluR6. The protein structural models were minimized for 20,000 steps of the conjugate gradient method by CHARMM27 [36] force filed in NAMD 2.13 package [37].

#### Covalent docking

The minimized structures of opto-mGluR6 and red-shift variants were used as receptors in molecular docking. The 11-cis-retinal 3D structure was retrieved from the ZINC database (https://zinc.docking.org/) and used as the ligand in molecular docking. The ligand alignment was used to attach the ligand to the receptor covalently. The receptor and ligand structural files, ligand atom indices, and the Lys317 catalytic residue were specified for ligand alignment. The standard and the flexible/rigid PDBQT files for the protein and covalent ligand structure, AutoGrid, and AutoDock parameter files were generated with MGLTools 1.5.6 [38]. The docking box was defined around Lys317 as the catalytic residue for covalent interaction. The genetic algorithm with 200 runs was used as the search algorithm. The “unbound model bound” entry in the AutoDock parameter files was edited to “unbound energy 0.0”. All other parameters were set to default values. All molecular griding and docking calculations were performed using AutoGrid and AutoDock 4.2 tools [39]. Complex structures of 11-cis-retinal with opto-mGluR6 and red-shift variants were shown with VMD1.9.3 [35]. The residues involved in these interactions and their interaction types were determined by LigPlot^+^ v.1.4 [40].

### Molecular biology

#### Cloning of Opto-mGluR6 and red-shift variants

A sequence of Opto-mGluR6 was obtained from the NCBI (Gene ID: JB414806.1). The ROM19, ROM18, ROM17, and Opto-mGluR6 sequences were synthesized and cloned into pAAV2.2-CMV-IRES-EGFP (AAV Helper-Free System, 240071, Agilent Technologies, USA) (S1 Fig in S1 File). The modified genetic structures were verified through both digestion and DNA sequencing to ensure their accuracy.

### In vitro assays

#### Stable HEK-GIRK cell line generation

HEK293 cell line was co-transfected by pcDNA3-Kir3.1 and pcDNA3-Kir3.2 (a kind gift from Dr. Terry Hébert, McIntyre Medical Sciences Building, Montreal, Canada) using the calcium phosphate precipitation method. 24 hours after transfection, the cells were treated with medium supplemented by 600 μg Geneticin (10131027, ThermoFisher Scientific). The medium was replaced every 2 days. After 10 days, a single-cell clone was generated by limited dilution and expanded by routine protocols.

#### Western blot

HEK-GIRK cells were harvested in lysis buffer (150 mM NaCl, 50 mM Tris, 1 mM EDTA and 1% Triton X-100 supplemented with protease inhibitor cocktail) and centrifuged at 10,000 x g for 10 min at 4ºC. For western blotting analysis, the sample protein concentration was determined using either the Bovine Serum Albumin (A7030, Sigma-Aldrich, Darmstadt, Germany) or the Bradford assay. Equivalent amounts of the proteins were subjected to SDS-PAGE on polyacrylamide gels and blotted onto Whatman nitrocellulose membranes (WHA10401170, Sigma-Aldrich). Blotted membranes were blocked for 1 hour in 5% Bovine Serum Albumin in Tris-buffered saline (10 mM Tris, 150 mM NaCl, pH 8.0) plus 0.1% Triton X-100 and incubated overnight at 4ºC with c-Myc (1/1000, ab19233, abcam, Cambridge, United Kingdom) and HA (1/1000, 11867423001, Roche, Basel, Switzerland) primary antibodies. Membranes were washed and incubated at room temperature for 2 hours with rabbit anti-mouse and goat anti-rabbit (1/5000, abcam, Massachusetts, USA) secondary antibodies. Bands were revealed with the ECL chemiluminescence detection system (ThermoFisher Scientific).

#### RNA expression of Opto-mGluR6 and red-shift variants

HEK-GIRK cells were seeded into a 6-well plate at a density of 1 × 10^6^ cells/well in 1ml of 10% Fetal Bovine Serum (FBS, 10099141, Gibco, Canyon, Australia) containing Dulbecco’s Modified Eagle Medium (DMEM, D5796-500 mL, Sigma-Aldrich). The next day, cells were transfected with AAV-CMV-IRES-EGFP, AAV-CMV-Opto-mGluR6-IRES-EGFP, AAV-CMV-ROM19-IRES-EGFP, AAV-CMV-ROM18-IRES-EGFP and AAV-CMV-ROM17-IRES-EGFP plasmids independently. 48 hours after transfection, total RNA was isolated using Tripure isolation reagent (11 667 157 001, Roche, Germany) and quantitative real-time PCR was performed with Opto-mgluR6 specific primer sets (Forward: ATTCTTCTCTTCGTGCTGTCCTG, Reverse: GGTGAGTGATGGCGTAGATAATGG). As an internal control, the housekeeping gene, glyceraldehyde phosphate dehydrogenase (GAPDH, QT01192646, Qiagen, Hilden, Germany) was used to normalize gene expression data. The reverse transcription and amplification were performed using the Qiagen One-Step RT-PCR kit (Qiagen Inc, Maryland, USA) with a LightCycler system (Roche, Penzberg, Germany). RT-qPCR conditions were as follows: Hot-start DNA polymerase was activated at 95°C for 5 min, followed by 35 cycles of (95°C for 30 s, 55°C for 30 s, and 72°C for 30 s) and 10 min at 72°C.

#### Protein expression of Opto-mGluR6 and red-shift variants

HEK-GIRK cells were seeded into a 24-well plate at a density of 2 × 10^5^ cells/well in 500 μl of 10% FBS-containing DMEM medium. The next day, cells were transfected by AAV-CMV- Opto-mGluR6-IRES-EGFP, AAV-CMV-ROM19-IRES-EGFP, AAV-CMV-ROM18-IRES-EGFP and AAV-CMV-ROM17-IRES-EGFP plasmids or pAAV-CMV-IRES-EGFP plasmid as control. 48 hours after transfection, sample cultures were fixed with 4% paraformaldehyde for 12 hours and immunostained by goat anti-melanopsin antibody (1/5000; AB-N38, Advanced Targeting Systems, Carlsbad, CA, USA). The Secondary antibody, donkey anti-goat IgG-R (1/200, sc-2094, Santa Cruz Biotechnology, Texas, USA) was applied for 2 hours at room temperature. The cell nuclei were stained by 4´, 6-diamidino-2-phenylindole dihydrochloride (DAPI, CAS 28718–90, Santa Cruz Biotechnology) for 2 min in the dark at room temperature. Slides were examined by an Axiophot Zeiss fluorescence microscope equipped with a 460 nm filter and 610 nm filter for DAPI and Rhodamin-labeled antibodies. Negative controls (only secondary antibodies) were also included in all experiments.

#### UV–Vis spectrophotometry of Opto-mGluR6 and red-shift variants

HEK-GIRK cells were seeded into a 100 mm cell culture plate at a density of 5 × 10^6^ cells/well in 10% FBS-containing DMEM medium. The next day, cells were transfected by AAV-CMV- Opto-mGluR6-IRES-EGFP, AAV-CMV-ROM19-IRES-EGFP, AAV-CMV-ROM18-IRES-EGFP and AAV-CMV-ROM17-IRES-EGFP plasmids or pAAV-CMV-IRES-EGFP plasmid as control. 48 hours later, cells were lysed with 1.5 ml ice-cold PBS containing protease and phosphatase inhibitors and regenerated with 10 μM all-trans-retinal (116-31-4, Sigma-Aldrich, Darmstadt, Germany). The lysates were frozen at −80 ◦C for 1 hour and thawed at room temperature. After 3 cycles of freeze and thawing, samples were centrifuged at 13,000 × g for 25 min. The supernatants were transferred to a new tube and labelled as the cytoplasmic fraction. The pellets were suspended with 0.75 ml membrane protein isolation buffer (20 mM Tris-HCl; 150 mM NaCl; 1 mM EDTA; 1 mM EGTA; and 1% Triton X-100, pH 7.5) containing protease and phosphatase inhibitors. The lysates were treated with ultrasonication briefly and centrifuged at 13,000 × g for 25 min. The supernatants were transferred to another new tubes and considered as the solubilized membrane fractions. Purified opsins were spectroscopically characterized using a Varian Cary 100 Bio spectrophotometer (Varian) in the 400–750 nm range.

#### Whole-cell patch-clamp recording

HEK-GIRK cells were transfected with AAV-CMV-IRES-EGFP, AAV-CMV-Opto-mGluR6-IRES-EGFP, AAV-CMV-ROM19-IRES-EGFP, AAV-CMV-ROM18-IRES-EGFP, and AAV-CMV-ROM17-IRES-EGFP plasmids. Cells were seeded on glass coverslips in 24-well plates at a density of 10^5 cells per well in 500 μL of 10% FBS medium. After 8 hours, cells were treated with 10% FBS medium containing 2 μM all-trans-retinal (Sigma-Aldrich, Darmstadt, Germany). Whole-cell patch clamp recordings were performed 48 hours post-transfection.

Solutions; • Bath Solution: 25 mM KCl, 102 mM NaCl, 1 mM MgCl2, 25 mM HEPES buffer, and 30 mM D-glucose (pH 7.4). • Pipette Solution: 129 mM K-Gluconate, 10 mM KCl, 10 mM HEPES, 4 mM MgATP, and 0.3 mM Na3GTP (pH 7.3).

Patch Clamp Setup; Borosilicate patch pipettes (1.2 mm O.D., 0.9 mm I.D. with inner filament, WPI, Sarasota, FL) were pulled using a PC10 two-stage vertical puller (Narishige, Tokyo, Japan) and filled with the intracellular solution. Pipettes with resistances of 3–5 MΩ were used for recordings. Data were collected using a Multiclamp 700B amplifier (Axon Instruments, Foster City, CA) equipped with a Digidata 1320 A/D converter (Axon Instruments, Foster City, CA). Signals were low-pass filtered at 10 kHz and digitized at 20 kHz.

Light Stimulation; Light sources of blue (λ = 473 nm), green (λ = 520 nm), orange (λ = 550 nm), and red (λ = 600 nm) were used to activate Opto-mGluR6, ROM17, ROM18, and ROM19, respectively. The light intensity at the end of the light guide was approximately ~1018 photons cm-2 s-1.

Voltage Clamp Protocol; The voltage clamp protocol for GIRK ion channel assays began with a hyperpolarizing voltage of -120 mV for 200 ms, followed by a 200 ms depolarizing ramp to 120 mV. Cells were maintained at a consistent holding potential, and light stimuli of 10–50 seconds were applied to measure both peak and steady-state currents. Data were analyzed using Clampfit 10.6 (Molecular Devices). All experiments were conducted at room temperature.

Data Analysis; Example recordings for each type of designed protein were presented, and summary data for both peak and steady-state currents were shown using box and whisker plots. The boxes represent the interquartile range (IQR), the lines inside the boxes indicate the median and the whiskers extend to the smallest and largest values within 1.5 times the IQR. Outliers are depicted as individual points beyond the whiskers.

#### Statistical analysis

Experiments were performed in at least three independent sets, and the number of replicates for each sample was at least three times in each experiment. Two-tailed Student’s t-test was used to evaluate the statistical significance of the data, and P values of less than 0.05 were considered to be statistically significant.

## Result

### Opto-mGluR6 modelling and red Opto-mGluR6 variants design

The wild-type model of opto-mGluR6 was selected based on the comparison between SWISS-MODEL and I-TASSER best models. The common structure between these results with the lowest RMSD value was chosen for further analysis, which was designed based on the rhodopsin (PDB ID: 4ZWJ: A) with 20.1 and 96.1% for identity and coverage, respectively. MolProbity Ramachandran analysis displayed 91.0% and 98.0% of all residues in favored and allowed regions, respectively (S2 Fig in S1 File), which showed a reasonable structural quality. The Molegro virtual docker depicted Lys317 as the chromophore binding site in the melanopsin model.

**Fig 1:**
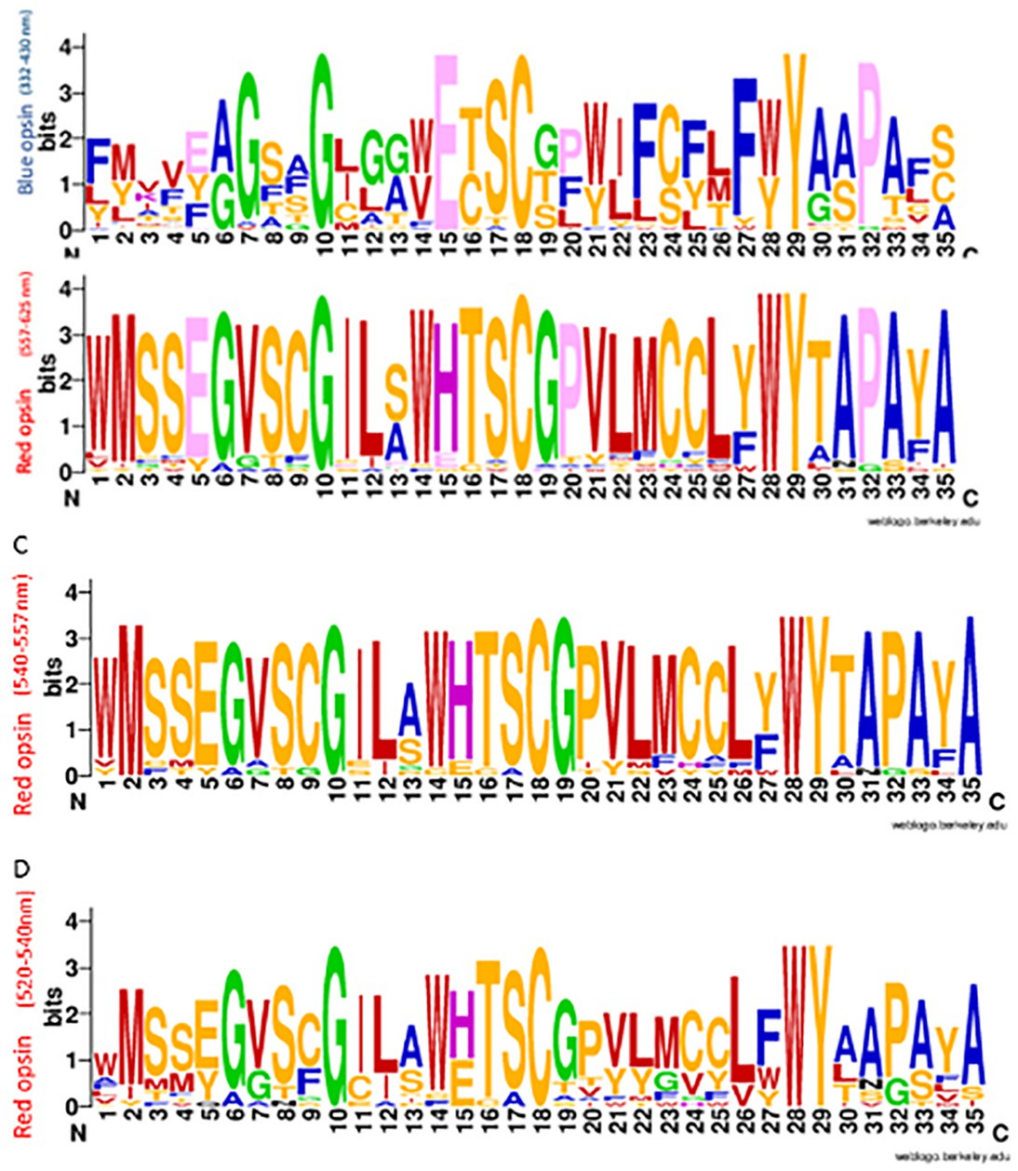
Logo of multiple alignments of retinal packet amino acids. (A) and (B) Multiple alignment logo of blue opsins with 332–340 nm and red opsins with 557–625 nm maximum absorption wavelength, respectively. (C) and (D) Multiple alignment logo of red opsins with 540–557 nm and 520–540 nm maximum absorption wavelength, respectively.

MSA of several melanopsins, rhodopsin, blue, green and red opsins are performed to show the roles of retinal binding pocket amino acids (amino acid sequence surrounding retinal in opsin) in shifting the maximum absorption of the opsins (S3 Fig in S1 File). Previous studies demonstrated that 35 amino acids of opsin are located in the retinal binding pocket [29]. The MSA of mentioned 35 amino acids in the blue opsins with 332–340 nm, red opsins with 557–625 nm, red opsins with 540–557 nm, and red opsins with 520–540 nm maximum absorption were shown in Fig 1. The results depicted conserved amino acids in this region with respect to the maximum absorption. Based on the MSA results, some of the amino acids that play critical roles in the absolute value of the maximum absorption of Opto-mGluR6 were selected and used to design red-shifted variants of Opto-mGluR6.

In this study, 3 mutant forms of opto-mGluR6 were assessed, denoted as ROM17, ROM18, and ROM19, each containing 17, 18, and 19 mutations, respectively. The mutations shared among ROM17, ROM18, and ROM19 include V75W, I76M, A123S, F126S, A146G, G149V, A150S, F152C, M157L, S223G, W224P, Y226V, F241M, V244C, F245C, S290T, and V314Y. Additionally, the F282Y mutation is present in ROM18 and ROM19, while A199S is specific to ROM19. Consequently, F282Y and A199S are highlighted as distinct mutant residues between ROM17, ROM18, and ROM19 in the accompanying FIG and elaborated upon in the legend.

Table 1 briefly represents retinal binding pocket amino acids and the best top 3 red-shift constructs. The selected predictions called as follows (Red Opto-mGluR6) ROM 19 (λmax = 557–625 nm), ROM18 (λmax = 540–557 nm) and ROM17 (λmax = 520–540 nm).

**Table 1:** Predicted red-shifted variants of Opto-mGluR6 and substituted amino acids in retinal binding pocket were depicted in bold, *italic*, and underlined format.

Construct	λmax (nm)	Amino acids	Mutation points
Opto-mGluR6	480	**VIAFYAGAFGIMAWETSCSWYLFVFLFWYSAPAVA**	-
ROM19	557–625	***WMSS*** **Y** ***GVSC*** **GI** ***LS*** **WETSC** ***GPV*** **L** ***MCC*** **L** ***Y*** **WY** ***T*** **APA** ***Y*** **A**	V75W, I76M, A123S, F126S, A146G, G149V, A150S, F152C, M157L, S223G, W224P, Y226V, F241M, V244C, F245C, S290T, V314Y.
ROM18	540–557	***WMSS*** **Y** ***GVSC*** **GI** ***L*** **AWETSC** ***GPV*** **L** ***MCC*** **LFWY** ***T*** **APA** ***Y*** **A**	V75W, I76M, A123S, F126S, A146G, G149V, A150S, F152C, M157L, S223G, W224P, Y226V, F241M, V244C, F245C, **F282Y**, S290T, V314Y.
ROM17	520–540	***WMSS*** **Y** ***GVSC*** **GI** ***L*** **AWETSC** ***GPV*** **L** ***MCC*** **LFWY** ***T*** **APA** ***Y*** **A**	V75W, I76M, A123S, F126S, A146G, G149V, A150S, F152C, M157L, **A199S**, S223G, W224P, Y226V, F241M, V244C, F245C, **F282Y**, S290T, V314Y.

**F282Y** and **A199S** are bold and underlined as distinct mutant residues between ROM17, ROM18, and ROM19 in the accompanying FIG and elaborated upon in the legend.

Mutations were implemented on the DNA sequence of Opto-mGluR6 by optimizing codon usage, and then their RNA secondary structures and the RNA minimum free energies (MFE) were predicted. The results revealed that the RNA secondary structure and MFE of ROM19, ROM18, and ROM17 were stable and similar with regard to the wild-type Opto-mGluR6 (S4 Fig and S1 Table in S1 File). Then protein structures of the designed red-shifted variants were generated based on the model of the wild-type opto-mGluR6.

#### 11-cis-retinal covalent docking with the Opto-mGluR6 and red-shift variants

The 11-cis-retinal as a chromophore attaches to the proteins, covalently, via a protonated Schiff-base [41]. The rhodopsin studies showed that the aldehyde group of the retinal is covalently bonded to the amino group of a lysine [42, 43]. So, the covalent docking of 11-cis-retinal with the Lys317 residue in the minimized structures of the wild-type and mutant variants of opto-mGluR6 was investigated. This structural configuration is consistent with X-ray structures, such as those available in PDBIDs 2Z73 and 1U19. It is also crucial to acknowledge that the ligands demonstrate flexibility during docking using AutoDock.

In the WT, ROM17, and ROM 18 docking, additional cis-isomerizations occur in carbons other than C11. The additional cis-isomerization facilitates the covalent binding of the end of 11-cis-retinol with Lys317.

**Fig 2:**
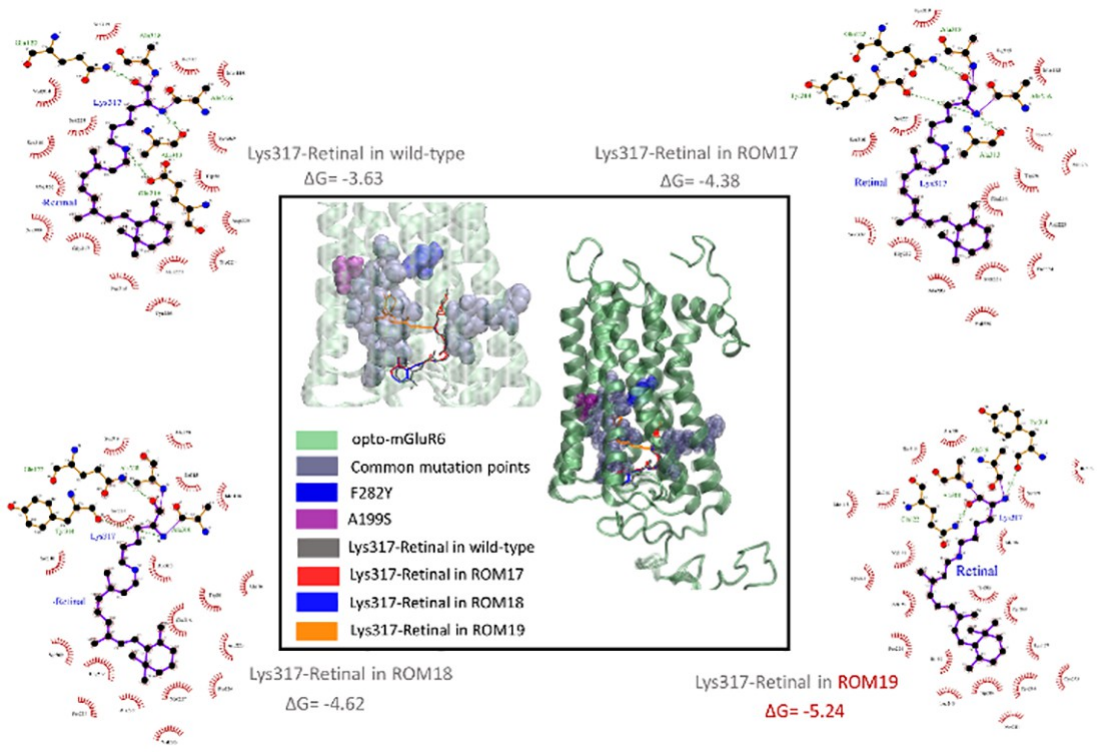
11-cis-retinal covalent docking with the wild-type (Opto-mGluR6) and red-shifted variants (ROM17, ROM18, and ROM19). Opto-mGluR6 3D structure and common mutation points, F282Y (especially for ROM18 and ROM19) and A199S (especially for ROM19) were shown in green, purple, blue, and magenta. The Lys317-retinal in interaction with wild-type, ROM17, ROM18, and ROM19 were depicted in brown, red, blue, and orange. In interaction diagrams, the protein residues involved in hydrophobic contacts and hydrogen bonds were represented in red-spoked arcs and green dotted lines, respectively.

The complex aligned structures, the interaction diagrams, and the docking binding free energy (ΔG) values of the covalent dockings are depicted in Fig 2. The interaction of 11-cis-retinal with ROM19 showed the lowest binding free energy (ΔG = −5.24 kcal/mol) and different binding modes compared to other red-shifted variants and opto-mGluR6. At the subsequence levels, 11-cis-retinal interacted with ROM18, ROM17, and the opto-mGluR6 with the binding free energies of -4.62, -4.38, and -3.63, respectively. These results indicated that the most stable interaction of 11-cis-retinal is for the ROM19 variant (Fig 2).

### Opto-mGluR6 and red-shift variants expression in HEK-GIRK cell line

**Fig 3:**
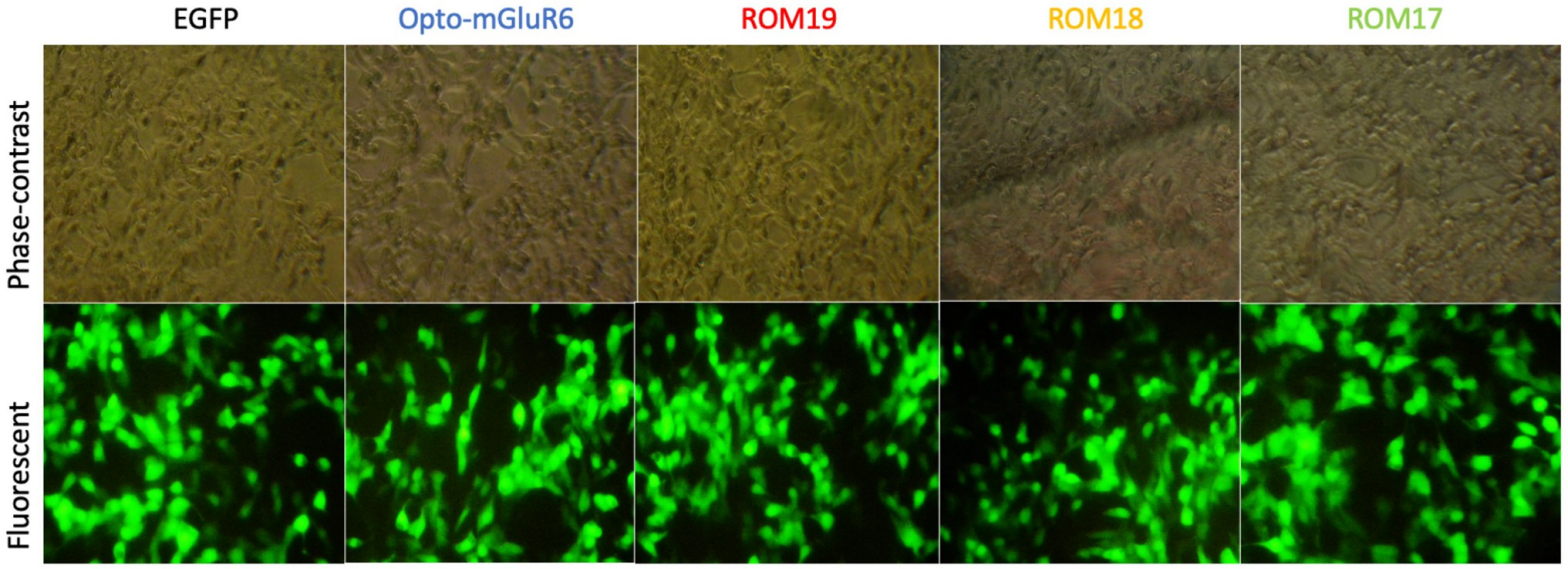
Photograph of transfected HEK-GIRK cell line. Top row: Phase contrast photograph of HEK-GIRK cells transfected by AAV-CMV-IRES-EGFP, AAV-CMV-Opto-mGluR6-IRES-EGFP, AAV-CMV-ROM19-IRES-EGFP, AAV-CMV-ROM18-IRES-EGFP and AAV-CMV-ROM17-IRES-EGFP plasmids. Bottom row: Fluorescent photograph of HEK-GIRK cells corresponding to the top row (Magnification: 200X).

**Fig 4:**
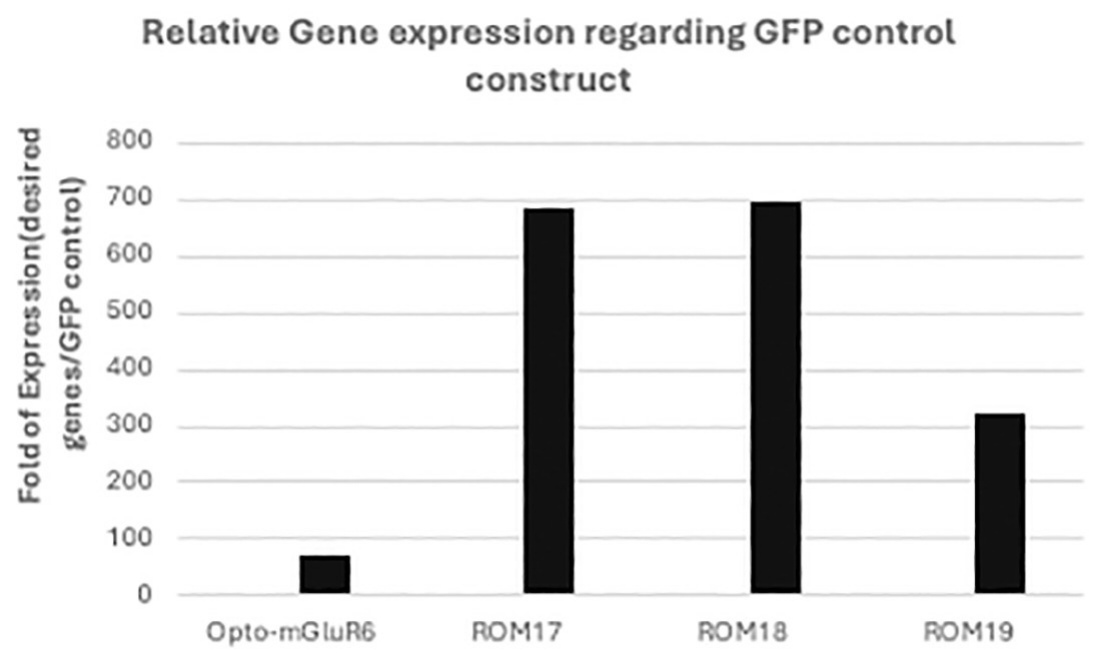
HEK293-GIRK cells were transfected by Opto-mGluR6 and red-shifted variants. RNA expression of Opto-mGluR6, ROM19, ROM18 and ROM17 were confirmed by real-time PCR.

**Fig 5:**
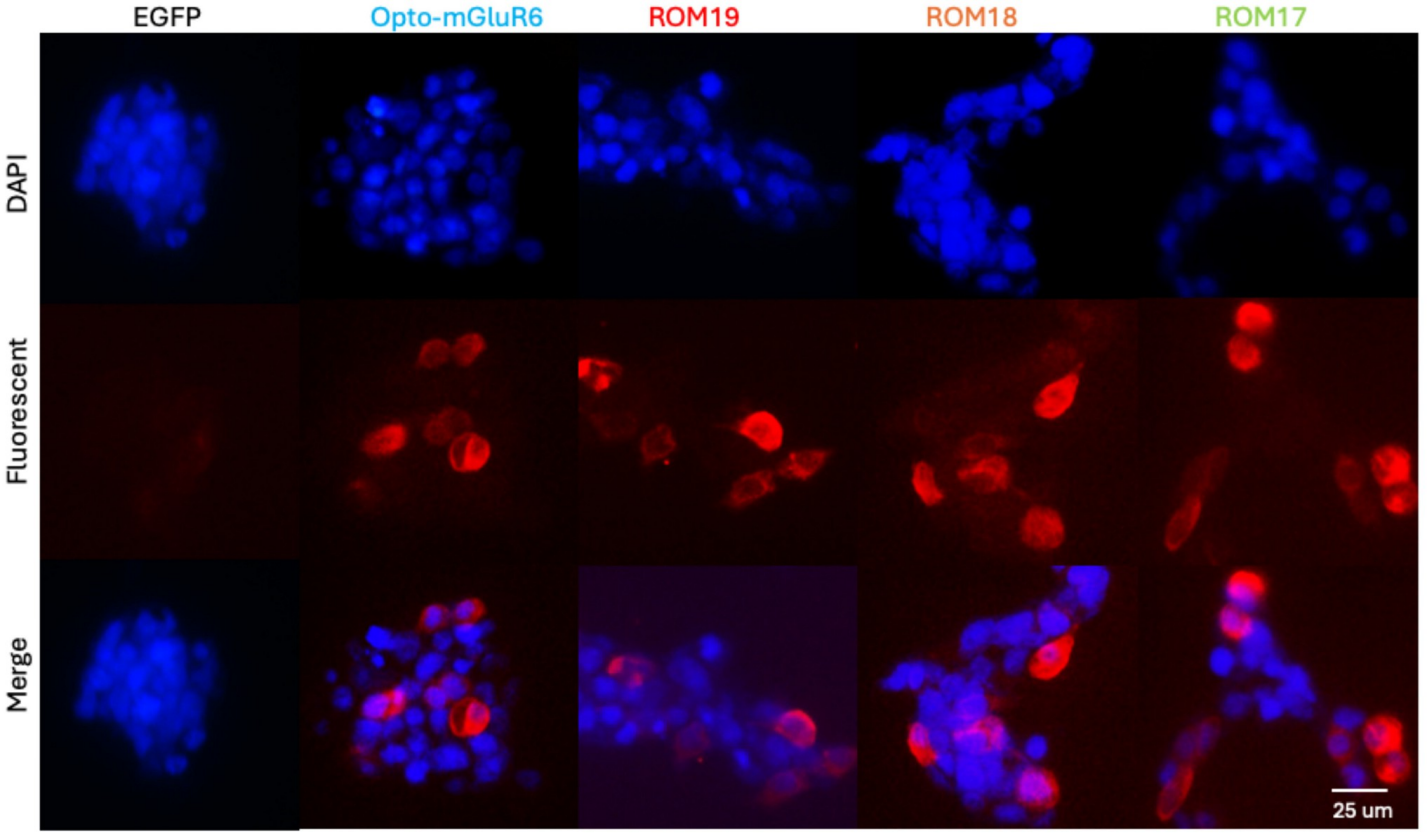
Melanopsin expression in the HEK-GIRK cell line transfected by Opto-mGluR6 and red-shifted variants. Top row: The nuclei of the cells were stained by DAPI. Middle row: The cells were immune stained by an anti-melanopsin antibody. Bottom row: Merge of DAPI and antibody. Magnification: 200X.

To investigate the expression and functionality of Opto-mGluR6 and its red-shifted variants, we generated HEK-GIRK cell lines stably expressing GIRK channels. The successful integration and expression of GIRK channels were first confirmed via Western blot analysis, as depicted in S5 Fig in S1 File. The clear presence of GIRK protein bands verified that the cell lines were appropriately prepared for further experiments involving Opto-mGluR6 and its variants. Following the establishment of these cell lines, the HEK-GIRK cells were transfected with plasmids encoding either Opto-mGluR6 or its red-shifted variants (ROM17, ROM18, and ROM19). Transfection efficiency was validated 48 hours post-transfection by monitoring GFP expression through fluorescence microscopy, as shown in Fig 3. The consistent and robust GFP signal across all cultures indicated a high transfection efficiency of approximately 90% across independent experiments, ensuring reliable expression of the constructs in the HEK-GIRK cells. To quantify the expression levels of the transfected genes, RNA was extracted from the cultures, and expression levels were assessed using real-time PCR, Fig 4. Interestingly, while all constructs showed significant upregulation of RNA transcripts, the Opto-mGluR6 construct exhibited a comparatively modest increase, showing a 71-fold increase in RNA transcripts. In contrast, the red-shifted variants demonstrated a much more substantial upregulation, with transcript levels increasing between 300 to 700-fold. This discrepancy in RNA expression levels suggests potential differences in transcriptional regulation or mRNA stability between Opto-mGluR6 and its red-shifted variants. The membranous localization of the expressed proteins was further assessed using fluorescence microscopy, as shown in Fig 5. Both Opto-mGluR6 and its red-shifted variants exhibited clear localization to the plasma membrane, which is essential for their function as light-sensitive opsins. The fluorescence images confirmed that, despite the differences in RNA expression levels, the proteins were effectively trafficked to the membrane, a critical step for their potential utility in optogenetic applications. These observations suggest that the variations in RNA expression do not adversely impact the correct localization of the opsins, although the functional implications of these differences warrant further investigation.

### UV–Vis spectrum of Opto-mGluR6 and red-shift variants

**Fig 6:**
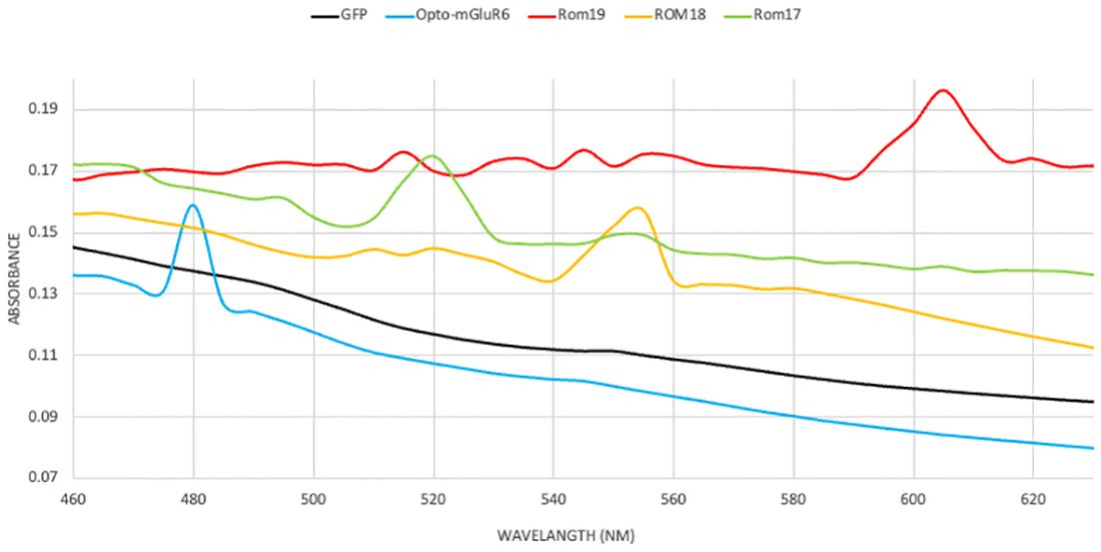
UV–VIS spectrophotometry of Opto-mGluR6 and red-shifted variants. The UV–Vis spectrum of purified Opto-mGluR6, ROM19, ROM18, and ROM17 showed absorption maxima at 480 nm, 520 nm, 550 nm, and 605 nm, respectively.

The spectral properties of Opto-mGluR6 and its red-shifted variants were examined using UV-Vis spectroscopy to characterize their photophysical attributes, which are crucial for their application in optogenetics. As shown in Fig 6, the UV-Vis absorption spectra of the purified opsins revealed distinct absorption maxima for each variant, correlating with their designed red-shifts. Opto-mGluR6, the original construct, exhibited an absorption maximum at 480 nm, characteristic of its intended spectral properties. The red-shifted variants, ROM19, ROM18, and ROM17, demonstrated progressive shifts in their absorption peaks, with maxima at 520 nm, 550 nm, and 605 nm, respectively. These shifts confirm the successful engineering of the opsins to extend their sensitivity to longer wavelengths of light, which is advantageous for in vivo applications where deeper tissue penetration by light is required. The distinct and predictable shifts in the absorption maxima indicate that the structural modifications introduced in the red-shifted variants effectively altered their chromophore environments, thereby adjusting their spectral sensitivities as designed. This broad range of absorption peaks allows for potential use in multi-wavelength optogenetic experiments, where different opsins can be activated independently using specific wavelengths of light. In summary, the data presented in Figs 3–6 provide a comprehensive overview of the successful expression, localization, and spectral characterization of Opto-mGluR6 and its red-shifted variants in HEK-GIRK cells. These findings lay a solid foundation for future functional studies to explore the optogenetic potential of these constructs in more complex biological systems.

### Opto-mGluR6 and red-shifted variants function in the HEK-GIRK cell line

**Fig 7:**
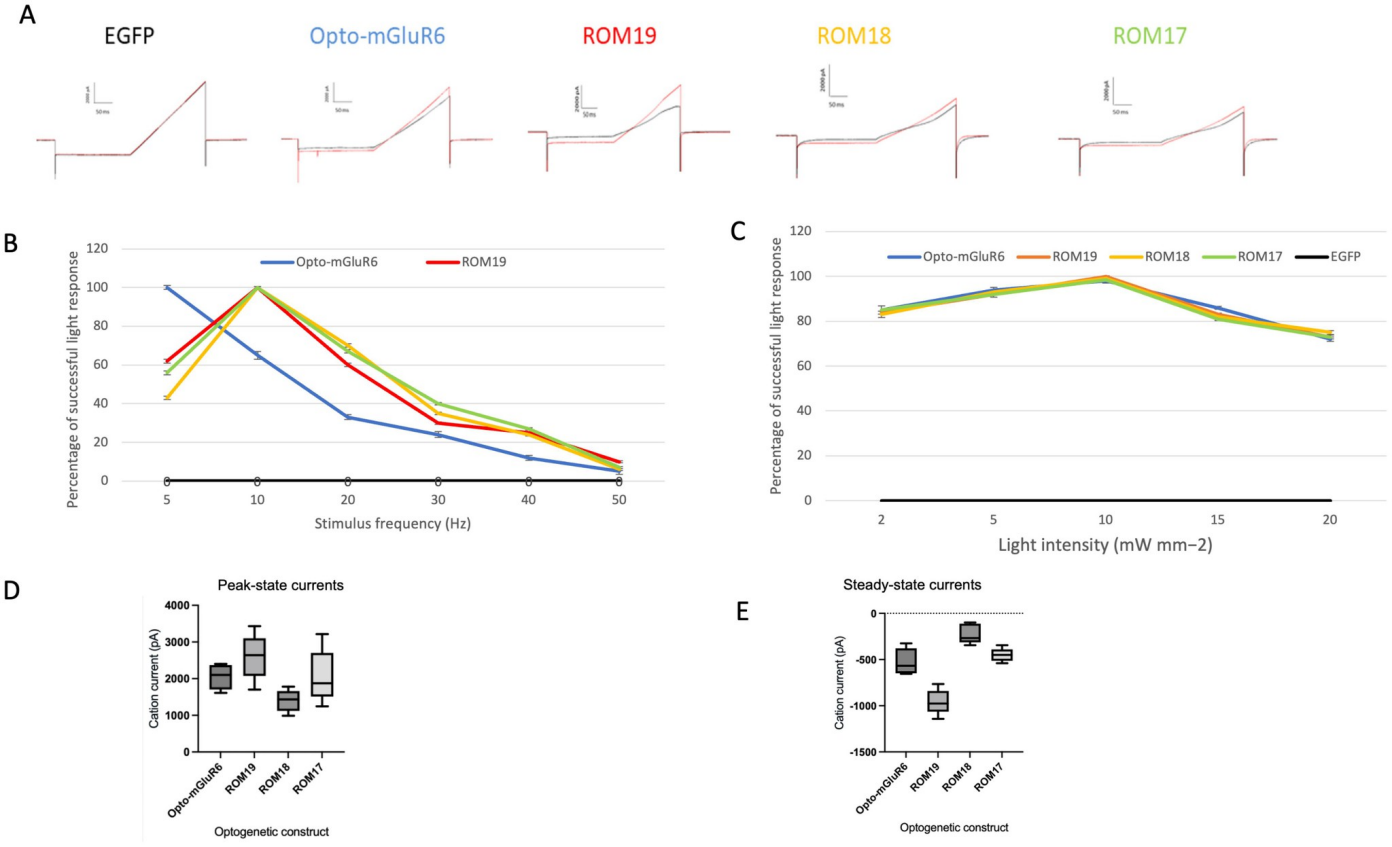
The light response of HEK-GIRK cells transiently transfected by Opto-mGluR6 variants. (A) Whole-cell voltage clamp from the HEK-GIRK cells to 200 ms voltage ramps between -120 mV and +120 mV in the dark (black line) and during light stimulation (red line). B) Summary data of mean current of Opto-mGluR6; ROM19, ROM18 and ROM17 evoked by 480-520-550-605 nm light over increasing light intensities (*n* = 5). (B) threshold of light response in cells transfected with Opto-mGluR6; ROM19, ROM18 and ROM17 by increasing light-pulse frequency. Prominent current responses were recorded at 5 Hz for Opto-mGluR6 and 10 Hz for red-shifted variants (n = 5). (C) Summary data of mean current of Opto-mGluR6, ROM19, ROM18, and ROM17 evoked by 480-520-550-605 nm light over increasing light intensities (*n* = 5). Opto-mGluR6 and red-shifted variants represent a maximum light response in the 10-mW mm−2 light intensity. (D) Box-and-whisker plot illustrating the distribution of cation current data for Opto-mGluR6 variants ROM19, ROM18, and ROM17, measured during the steady-state phase. The plot shows that ROM19 exhibits a broader range of photocurrent values with a median of -915 pA, compared to medians of -267 pA and -487 pA for ROM18 and ROM17, respectively. ROM19 recorded the highest peak current at -1142 pA and displayed a red-shifted activation profile, particularly in comparison to ROM18 and ROM17, indicating superior performance. The interquartile ranges further highlight the consistently higher photocurrent in ROM19. These data suggest that ROM19 is a more efficacious variant in terms of photocurrent generation and activation shift under the experimental conditions. (E) Box-and-whisker plot illustrating the distribution of cation current data for Opto-mGluR6 variants ROM19, ROM18, and ROM17, measured during the peak-state phase. The plot indicates that ROM19 has a broader range of photocurrent values with a median of 2450 pA, reflecting a strong and consistent response. In contrast, ROM18 has a slightly lower median of 1546 pA, and ROM17 a median of 1780 pA, both with narrower interquartile ranges. ROM19 also exhibits the highest peak current at 3432 pA, indicating a significantly greater capacity for photocurrent generation compared to ROM18 and ROM17. These findings underscore ROM19’s enhanced performance, particularly in achieving higher peak currents and a wider range of activation, positioning it as a potentially more effective variant under the experimental conditions.

The activities of the Red Opto-mGluR6 variants were determined using an electrophysiological screening of HEK-GIRK cells. The plasmids encoding each of the Red Opto-mGluR6 variants were transiently transfected into independent HEK-GIRK cell cultures. Red Opto-mGluR6 variants were activated by light, coupling light stimuli to G-protein signaling (Fig 7A). Opto-mGluR6 induced GIRK channel activation when stimulated by blue light at 5 Hz. However, light frequencies of 10 Hz or greater did not evoke greater current amplitudes in the HEK-GIRK cells transfected with Opto-mGluR6 (Fig 7B). At 5 Hz, cells fired 100% of the action potentials at 480 nm, while at 10 Hz, they typically fired in response to ~65% of the light pulses. The response was reduced to 33%, 24%, 12%, and 5% at 20 Hz, 30 Hz, 40 Hz, and 50 Hz, respectively (Fig 7B). ROM19, ROM18, and ROM17 induced GIRK channel activation when stimulated by 10 Hz red, orange, and green lights, respectively. However, prominent current responses were not recorded at 5 Hz or at frequencies of 20 Hz and above (Fig 7B). At 10 Hz, ROM19, ROM18, and ROM17-transfected HEK-GIRK cells fired 100% of the action potentials at 605, 550, and 520 nm, respectively. Current response decreased to approximately 65%, 35%, 25%, and 10% at 20 Hz, 30 Hz, 40 Hz, and 50 Hz. All Opto-mGluR6 and red-shifted variants exhibited maximum light response at 10 mW mm^−2^ light intensity (Fig 7C). Lower responses were observed with light intensities below and above 10 mW mm^−2^.

The analysis of cation current responses for Opto-mGluR6, ROM19, ROM18, and ROM17, as depicted in the box-and-whisker plots (Fig 7D and 7E), reveals distinct differences in both steady-state and peak-state currents across the variants. Opto-mGluR6 serves as the baseline control in this study, providing a reference point for comparing the performance of the variants. The median photocurrent for Opto-mGluR6 was -643 pA, with an interquartile range (IQR) from -700 pA to -567 pA. In the steady-state phase (Fig 7D), ROM19 exhibited the highest photocurrent values among the variants, with a median of -915 pA and a broad IQR ranging from -1142 pA to -764 pA. ROM19 also reached the highest maximum current of -1142 pA, indicating its superior photocurrent efficiency. In comparison, ROM18 had a median photocurrent of -267 pA with an IQR from -345 pA to -98 pA, reflecting a narrower range of photocurrent values. The highest observed current for ROM18 was -254 pA, which is significantly lower than the peak current observed for ROM19. ROM17 displayed a median photocurrent of -487 pA, with an IQR ranging from -987 pA to -254 pA. The maximum current for ROM17 was -987 pA, also lower than ROM19’s peak.

In the peak-state phase (Fig 7E), ROM19 demonstrated a notably broader range of photocurrent values, with a median of 2450 pA and an IQR spanning from 1800 pA to 2768 pA. The maximum photocurrent observed for ROM19 was 3432 pA, underscoring its superior capacity for generating high levels of photocurrent. In contrast, ROM18 exhibited a lower median photocurrent of 1546 pA, with an IQR from 1254 pA to 1780 pA. Although ROM18 reached a peak current of 1780 pA, its overall performance was less consistent compared to ROM19, as indicated by its narrower distribution of photocurrent values. ROM17, while displaying the lowest median photocurrent among the three variants at 1780 pA, had an IQR ranging from 1244 pA to 3214 pA. The maximum photocurrent for ROM17 was 3214 pA, suggesting some potential for high output, though its performance remained less consistent compared to ROM19. Overall, the data indicate that ROM19 outperforms ROM18 and ROM17 in terms of both peak photocurrent and response consistency. The enhanced photocurrent generation and broader activation range observed in ROM19, coupled with its red-shifted activation, suggest that it is a more effective variant for achieving high and reliable cation currents, making it a superior choice for optogenetic applications (Fig 7D and 7E).

Control EGFP-expressing cells did not respond to blue, green, orange, or red-light stimulation (S6 Fig in S1 File).

## Discussion

In this study, protein structure prediction was performed using homology modelling and fold recognition methods via the SWISS-MODEL and I-TASSER webservers, respectively. SWISS-MODEL is a fully automated server for protein structure homology modelling, while I-TASSER has been consistently ranked as the No. 1 server for protein structure prediction in recent CASP (Critical Assessment of Structure Prediction) experiments, including CASP7 through CASP15.

As outlined in the methods section, the structure with the lowest Root Mean Square Deviation (RMSD) value [44], indicating the highest similarity between the two methods, was selected for further analysis. It’s noteworthy that template selection was not solely based on sequence identity or similarity, although the chosen template exhibited over 20% identity and 96% coverage.

Moreover, the MolProbity Ramachandran analysis indicated reasonable structural quality for the selected model, reinforcing its suitability for subsequent analyses.

Three mutant forms of opto-mGluR6 were assessed, denoted as ROM17, ROM18, and ROM19, each containing 17, 18, and 19 mutations, respectively. The mutations shared among ROM17, ROM18, and ROM19 include V75W, I76M, A123S, F126S, A146G, G149V, A150S, F152C, M157L, S223G, W224P, Y226V, F241M, V244C, F245C, S290T, and V314Y. Additionally, the F282Y mutation is present in ROM18 and ROM19, while A199S is specific to ROM19. Three red-shifted variants of Opto-mGluR6, ROM19, ROM18 and ROM17 were pursued, and their expression and function in the HEK-GIRK cell line were confirmed (Figs 1–5). The ROM17, ROM18 and ROM19 red-shift variants responded strongly to green (λ = 520 nm), orange (λ = 550 nm), and red (λ = 605 nm) lights at 10 Hz frequency and 10 mW mm−2 light intensity. ROM19 showed greater photocurrents and more red-shifted activation compared to ROM18 and ROM17 (Figs 6 and 7).

Optogenetic tools for vision restoration have had significant shortcomings to date. The microbial opsins, Channelrhodopsin and Halorhodopsin, and the light-engineered mammalian receptors respond rapidly to the light and, therefore, should support “refresh rates” of sufficient speed for vision in motion, but they require intense light’s rays that may bring about the risk of damage to the retina cells [45]. Previous studies showed that ectopic melanopsin in retinal ganglion cells could be activated at low light intensities but, it culminated to poor temporal resolution with slow kinetic [24]. In 2015, Michiel van Wyk et al., recommended Opto-mGluR6 as a light-activated metabotropic receptor to amplify light signals by the G-protein-coupled signaling cascade of mGluR6 [16]. Opto-mGluR6 was over three times more responsive to light signals than ChR2 [46] as like as melanopsin does [24]. The temporal property of melanopsin has been improved through Opto-mGluR6 toward faster kinetics. The new variants of Opto-mGluR6 were introduced in 2022, and their function in restoring vision at the retinal, cortical, and behavioural levels was approved [47].

Rhodopsin is used as an optogenetic tool in bipolar cells or RGCs. It is native to the retina, far more sensitive than other optogenetic treatments and potentially safer for human applications. However, his opsin requires chromophore recycling in order to provide enough 11-cis retinal, which is necessary for phototransduction [48]. Opto-mGluR6 also has the advantage of being resistant to bleaching compared to Rhodopsin. Also, in comparison with the microbial opsins, as the strange protein molecules, eyesight establishment in blindness by the cognate genes of unmuted natural proteins such as melanopsin, rhodopsin, or cone opsins decreases the likelihood of triggering an immune response or the subsequent necessity for localized or systemic immune suppression [22].

Endogenous chromophores strongly absorb blue light and induce photochemical damage in the sensory retina. We introduced new red-shifted variants of Opto-mGluR6. Bioinformatics studies were recruited to find amino acid sequences of different mammalian opsins from data banks. Multiple alignments of the sequences against melanopsin were performed. Three red-shifted constructs of Opto-mGluR6 with estimated of activation spectrum- ROM 19 (λmax = 557-625nm), ROM18 (λmax = 540–557 nm) and ROM17 (λmax = 520–540 nm) were predicted (Figs 1 and 2).

Multiple alignments of sequences of melanopsins, rhodopsin, blue, green and red opsins demonstrated the roles of retinal binding pocket amino acids in maximum absorption and excitation of the opsins. The differences in the amino acid sequences of the retinal binding pocket are associated with an altered environment for the chromophore that gives rise to shifts in the maximum absorption spectrum [29, 49]. As a result of the so-called opsin shift effect—corresponding to 560 nm for the red cone pigment 530 nm for the green cone pigment, and 420 nm for the blue cone pigment [50, 51]. In 2008, VChR1 was introduced with maximum excitation red-shifted ~70 nm compared with ChR2. It was demonstrated that four residues of the retinal Schiff base binding pocket contribute to these absorption differences [18].

UV–Vis spectrum of purified opsins represented an increased maximum absorbance from 480 nm, corresponding to Opto-mGluR6, to 605, 550 and 520, corresponding to ROM19, ROM18 and ROM17, respectively (Fig 6). We confirmed that maximum absorbance depends on retinal banding packet amino acid. Previous studies also confirmed that specific amino acids at the retinal binding site, as well as their interactions with the retinal chromophore and surrounding water molecules, modulate the spectral tuning of visual opsins [52, 53]. Rhodopsin’s absorbance spectra of three Nymphalini butterfly species demonstrated the effects of retinal pocket amino acid substitutions on rhodopsin spectral tuning across 500 million years of evolution [54]. So far, different red-shifted variants of Channelrhodopsin, such as ReaChR, VChR1 and C1V1(E122T), have been introduced [18, 20]. In 2008, VChR1, from Volvox carteri, displayed maximum excitation at 589 nm, with a redshift of about 70 nm compared with ChR2, but its current kinetics were slower than those of ChR2 [18]. In 2011, an *M*. *viride* channelrhodopsin, MChR1, was introduced as a more red-shifted variant compared to VChR1 with pH-independent spectral sensitivity. Its current kinetics is significantly faster than VChR1, and it shows lower inactivation than VChR1 when stimulated with light of the same wavelength [55]. In 2014, channelrhodopsin CnChR1, from the species *Chlamydomonas noctigama*, with 660 nm far-red-light photocurrents with the nickname “Chrimson” was introduced. Chrimson is 45 nm more red-shifted than any other previously known channelrhodopsin [21].

The most significant cell response was observed when Opto-mGluR6 was activated by 480 nm light at a frequency of 5 Hz. Light frequencies of 10 Hz or greater produce a lower response in Opto-mGluR6-infected cells. This aligns with findings from other studies, where optogenetic tools are sensitive to specific wavelengths and frequencies for optimal activation. For instance, the original channelrhodopsin (ChR2) exhibits maximal activation at blue light (approximately 470 nm) and is often used at similar frequencies to achieve the desired cellular responses [56–59]. Meanwhile, red-shifted variants ROM19, ROM18, and ROM17 represented the greatest cell response by 605, 550, and 520 nm light at 10Hz, respectively. Lower and higher frequencies induced lower cell response (Fig 7B). This characteristic is particularly advantageous for in vivo applications, as red and near-infrared light penetrates deeper into biological tissues compared to blue light [21]. Studies on other red-shifted optogenetic tools, such as Chrimson and ReaChR, support these findings. Previous research showed that 90% of VChR1-transfected cells fired action potential at up to 10 Hz, while cell response was reduced by increasing the light frequency [18]. As a red-shifted variant of channelrhodopsin, C1V1, a chimaera of ChR1 and ChR2, demonstrated the maximum cell response at 545 nm at 5Hz, and cell response was reduced by increasing the light frequency [60]. Chrimson, a far-red-shifted channelrhodopsin, exhibited maximal cell response up to 20 Hz, with diminishing responses at higher frequencies [21].

All Opto-mGluR6 and red-shifted variants exhibited maximal light response at the 10 mW mm^−2^ (~1019 photons s-1 cm-2) light intensity. Light intensities below and above 10 mW mm^−2^ induced a lower light response (Fig 7C). ReaChR, an engineered chimeric variant of VChR1, exhibited maximum cell response when stimulated with 10 mW/mm^2^ of 590 nm light [20]. For C1V1, maximum cell response occurred at 2.5 mW/mm^2^ of 545 nm light [60]. According to a previous study, the level of light intensity required to stimulate Opto-mGluR6-expression in HEK293-GIRK cells was approximately ~1018 photons s-1 cm-2 [25], which was found to be consistent with the intensity of the red-shifted variants(~1019 photons s-1 cm-2) introduced by our lab.

Opto-mGluR6, ROM19, ROM18, and ROM17 represented great photocurrent in HEK-GIRK cells. ROM19 showed greater photocurrent and red-shifted activation compared to ROM18 and ROM17 (Fig 7D). ROM19 represents red-shift activation. It was designed based on native opsins such as AGI55881.1 LWS opsin [Aristostomias scintillans] and red-sensitive cone opsin [Xenopus laevis].

The cellular response patterns of optogenetic-transfected cells exhibited hyperpolarization. This phenomenon was also observed in a previous study, where hyperpolarization occurred in HEK293-GIRK cells engineered to express Opto-mGluR6 [25]. OPN1MW-mGluR6 and melanopsin were able to activate the GIRK currents by stimulating G-protein signaling in HEK293-GIRK cells [47, 61].

Overall, the introduced Opto-mGluR6 variants provide an innovative approach to studying red-shift optogenetic tools in vitro and in vivo. Through the reduction in the scattering of lower-energy photons, these opsins will be able to perform combinatorial control experiments with deeper penetration of light in in *vivo* applications.

## Supporting information

S1 File This document includes supplementary figures and analyses related to the study. S1 Fig shows a schematic representation of the pAAV-CMV-IRES-EGFP-Opto-mGluR6 vector, demonstrating the cloning of the Opto-mGluR6 gene using EcoRI and XhoI restriction enzymes. S2 Fig presents the Ramachandran plot analysis of the wild-type opto-mGluR6 model, highlighting favored and allowed regions. S3 Fig includes multiple sequence alignments of melanopsins, rhodopsin, and other opsins, focusing on amino acids involved in retinal binding and absorption shifts. S4 Fig provides RNA secondary structures of Opto-mGluR6 and related constructs ROM19, ROM18, and ROM17. S5 Fig displays an immunoblot for GIRK channel expression in HEK-GIRK cells, while S6 Fig shows the light response of these cells, transiently transfected with pAAV-CMV-IRES-EGFP, under different light stimulations in a whole-cell voltage clamp. S1 Table shows RNA minimum free energy prediction for optogenetic constructs.
(DOCX)

S1 Raw images (PDF)
