# Correction: Engineered red Opto-mGluR6 Opsins, a red-shifted optogenetic excitation tool, an in vitro study

**DOI:** 10.1371/journal.pone.0321044

**Published:** 2025-03-25

**Authors:** Hoda Shamsnajafabadi, Zahra-Soheila Soheili, Mehdi Sadeghi, Shahram Samiee, Pouria Ghasemi, Mohammad Ismail Zibaii, Hamid Gholami Pourbadie, Hamid Ahmadieh, Ehsan Ranaei Pirmardan, Najmeh Salehi, Dorsa Samiee, Ali Kashanian

The Acknowledgment section for this paper is not indicated. The correct Acknowledgment section is as follows: The authors would like to extend their gratitude to Professor Fabio Benfenati for his assistance in allowing them to perform the Western blot at his laboratory in the Italian Institute of Technology, Genova (Italy). They would also like to express their sincere appreciation to Dr. Terry Hebert from the McIntyre Medical Science Building, Montreal, Canada, for generously providing them with the pcDNA-Kir3.1 and pcDNA-Kir3.2 plasmids. The valuable contributions of Professor Benfenati and Dr. Hebert significantly facilitated the progress of this study.
