# Supplementary material for: Engineered red Opto-mGluR6 Opsins, a red-shifted optogenetic excitation tool, an in vitro study
Source: PLoS One. 2024 Oct 24;19(10):e0311102. doi: 10.1371/journal.pone.0311102 (PMC11500960; doi:10.1371/journal.pone.0311102)
Supplement: S1 File — S1 Fig shows a schematic representation of the pAAV-CMV-IRES-EGFP-Opto-mGluR6 vector, demonstrating the cloning of the Opto-mGluR6 gene using EcoRI and XhoI restriction enzymes. S2 Fig presents the Ramachandran plot analysis of the wild-type opto-mGluR6 model, highlighting favored and allowed regions. S3 Fig includes multiple sequence alignments of melanopsins, rhodopsin, and other opsins, focusing on amino acids involved in retinal binding and absorption shifts. S4 Fig provides RNA secondary structures of Opto-mGluR6 and related constructs ROM19, ROM18, and ROM17. S5 Fig displays an immunoblot for GIRK channel expression in HEK-GIRK cells, while S6 Fig shows the light response of these cells, transiently transfected with pAAV-CMV-IRES-EGFP, under different light stimulations in a whole-cell voltage clamp. S1 Table shows RNA minimum free energy prediction for optogenetic constructs. (DOCX) [file pone.0311102.s001.docx]

**Supplementary files**


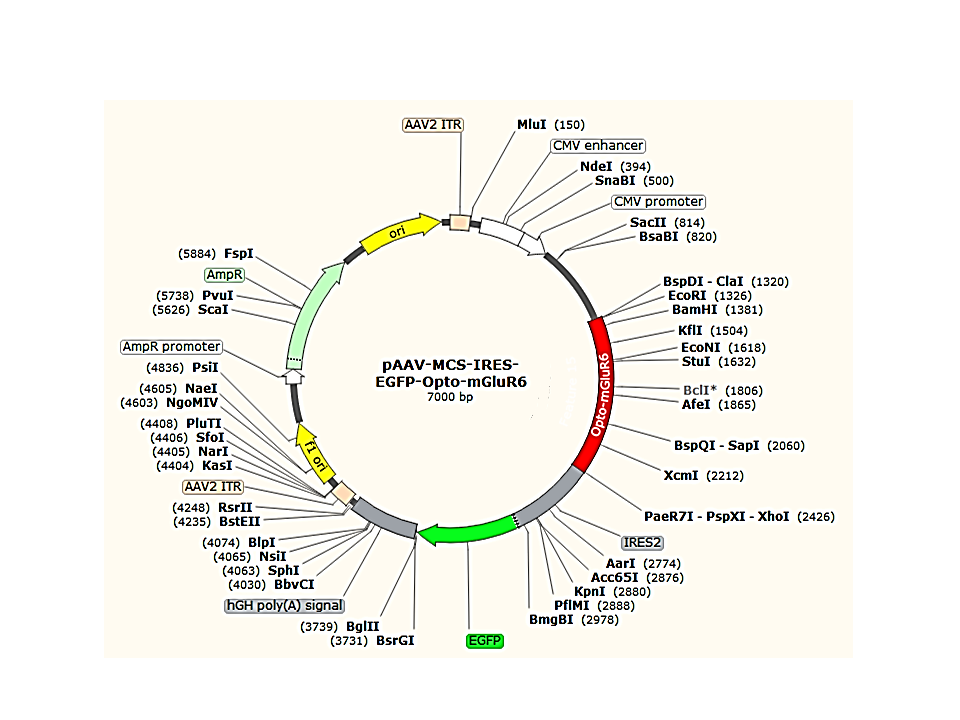


Fig S1. Schematic representation of pAAV-CMV-IRES-EGFP-Opto-mGluR6 vector. Opto-mGluR6 gene was cloned into pAAV-CMV-IRES-EGFP vector by EcoRI and XhoI restriction enzymes.


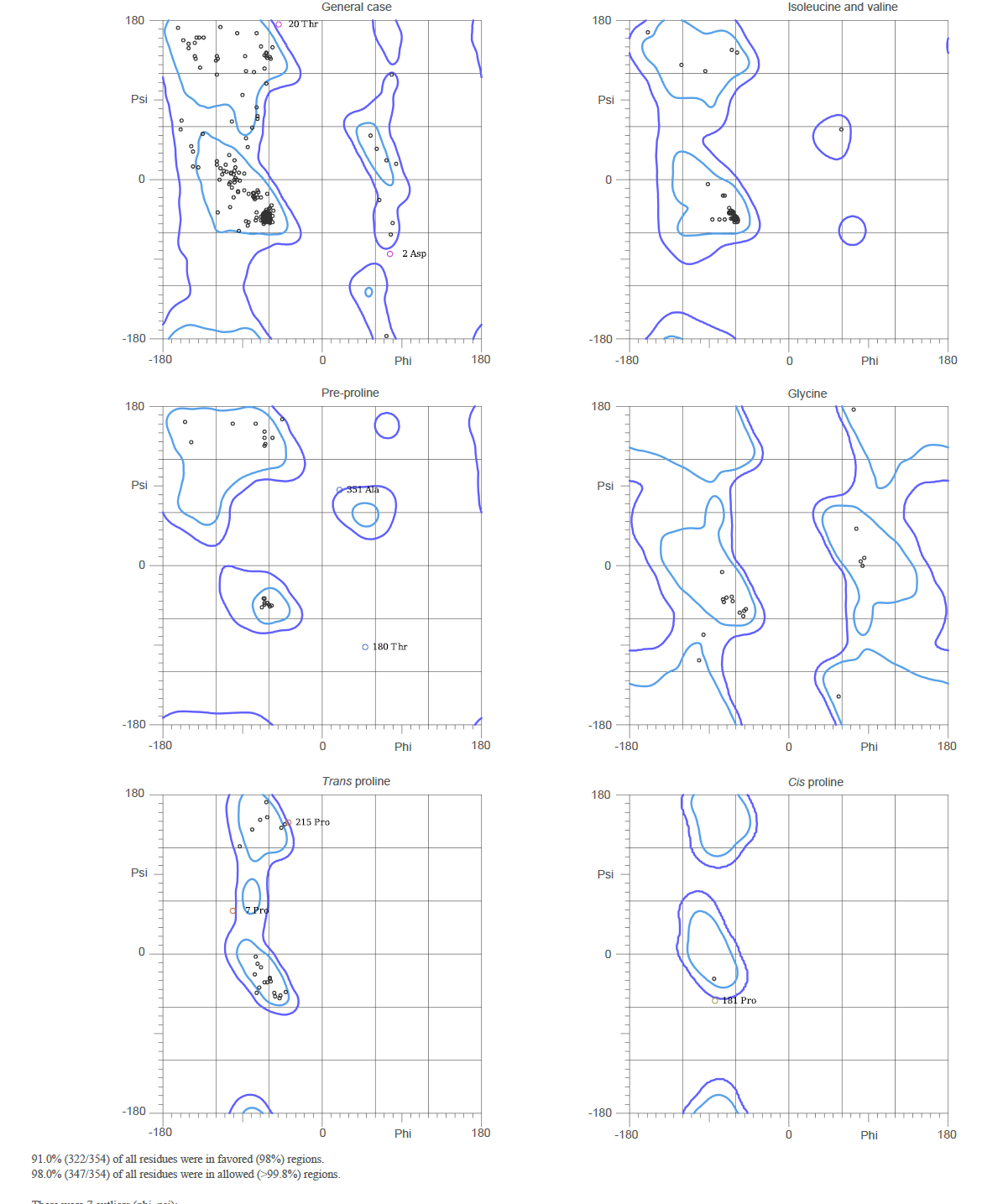


Fig S2. The Ramachandran plot analysis of the wild-type model of opto-mGluR6. The favored and allowed regions were shown in light and dark blue lines, respectively.


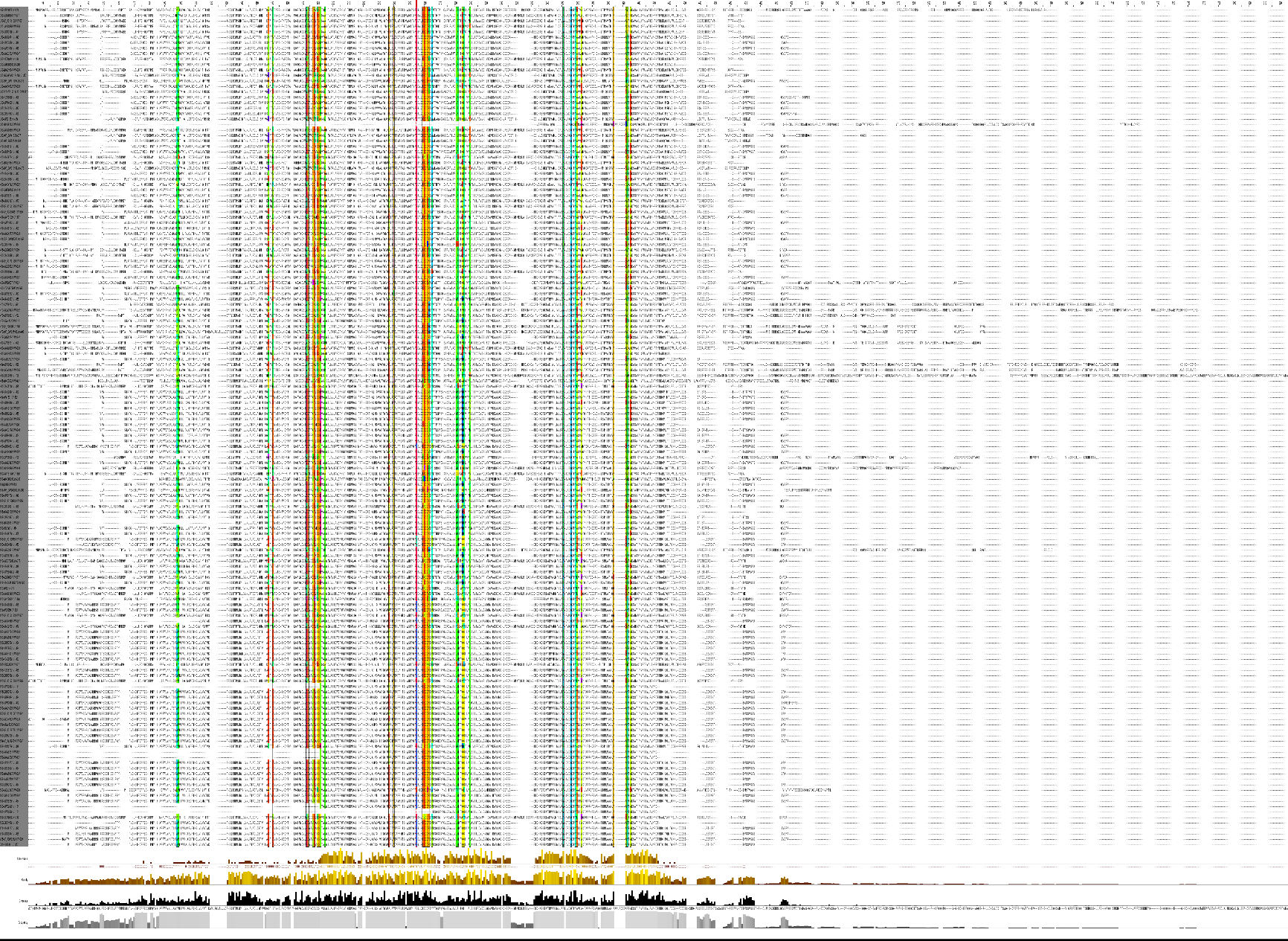


Fig S3. Multiple sequence alignments of several natural melanopsins, rhodopsin, blue, green and red opsins represent the role of 35 retinal binding pocket amino acids (high-lighted amino acids) in shifting the absorption maximum of the opsins.


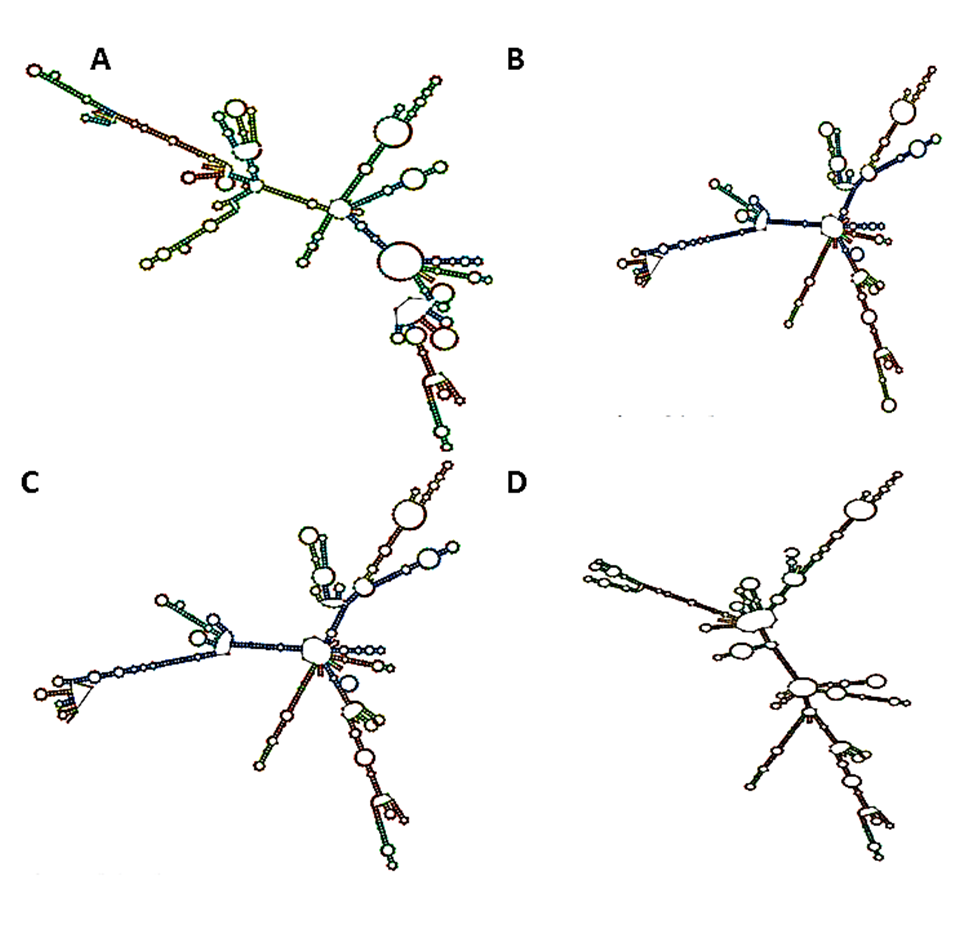


Fig S4. RNA secondary structure. A, B, C and D: RNA secondary structure of Opto-mGluR6, ROM19, ROM18 and ROM17.


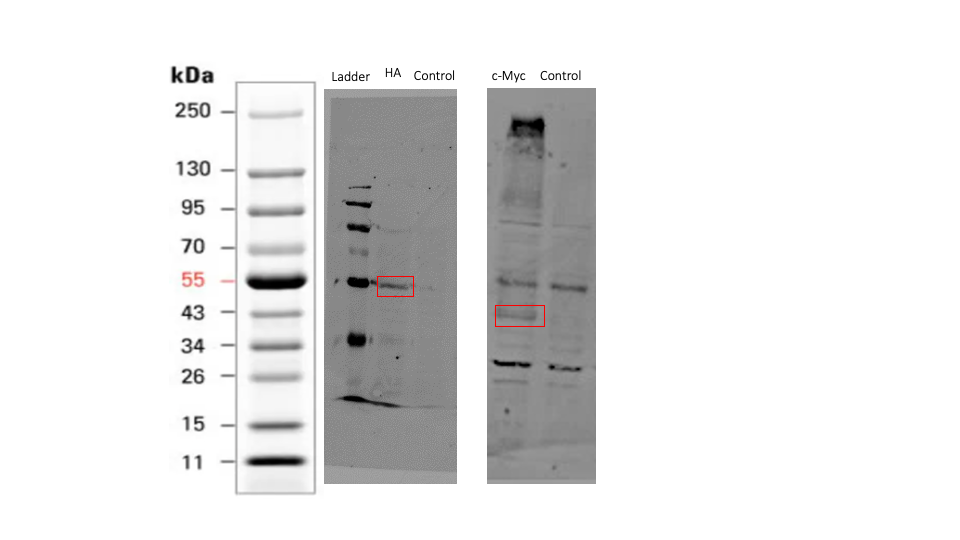


Fig S5. Immunoblot of GIRK channels expression in HEK_GIRK cells. HEK293 cells stably express HA-tagged GIRK1(56 kDa) and c-Myc-tagged GIRK2 (48.5 kDa) channels.

The red rectangle represents the related band on the gel.


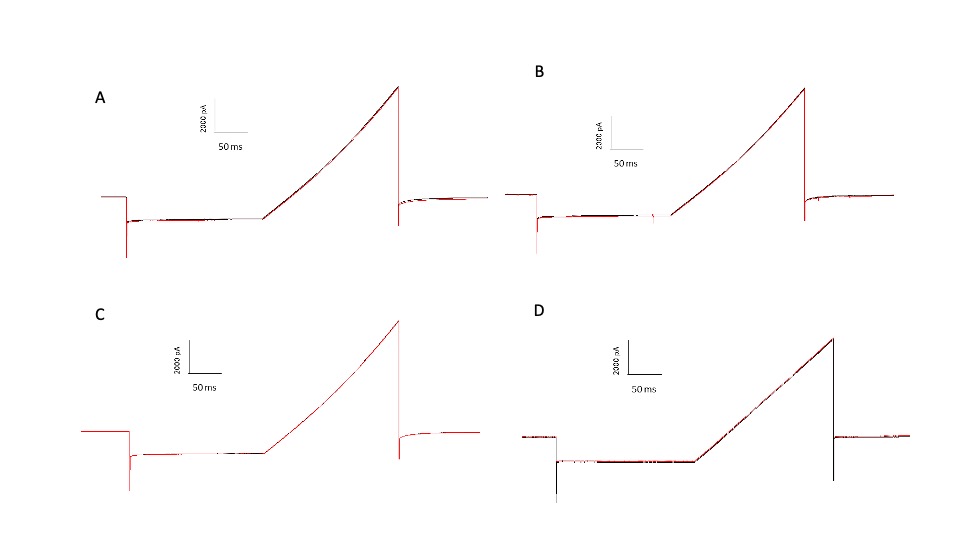


Fig S6. The light response of HEK-GIRK cells transiently transfected by pAAV-CMV-IRES-EGFP. A, B, C and D) Whole-cell voltage clamp from the by HEK-GIRK cells to 200 ms voltage ramps between -120 mV and +120 mV in the dark (black line) and during blue, green, orange, red light stimulation (red line). EGFP-expressing cells did not respond to blue, orange, or green lights.

| Table S1. RNA minimum free energy prediction for optogenetic constructs | |
| --- | --- |
| Optogenetic construct | minimum free energy(kcal/mol) |
| Opto-mGluR6 | -343.90 |
| ROM 19 | -344.10 |
| ROM18 | -342.20 |
| ROM17 | -342.90 |
