# Supplementary material for: Engineered red Opto-mGluR6 Opsins, a red-shifted optogenetic excitation tool, an in vitro study
Source: PLoS One. 2024 Oct 24;19(10):e0311102. doi: 10.1371/journal.pone.0311102 (PMC11500960; doi:10.1371/journal.pone.0311102)
Supplement: S1 Raw images — (PDF) [file pone.0311102.s002.pdf]

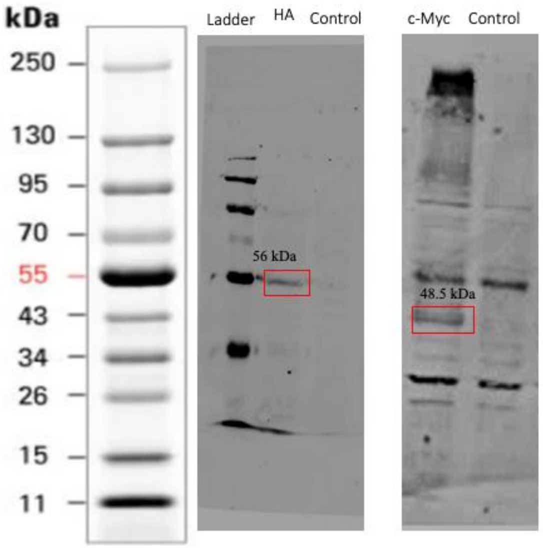


Fig S5. Immunoblot of GIRK channels expression in HEK_GIRK cells. HEK293 cells stably express HA-tagged GIRK1(56 kDa) and c-Myc-tagged GIRK2 (48.5 kDa) channels. The red rectangle represents the related band on the gel. The image was captured using the chemiluminescence method.
